# Supporting post-pandemic recovery: a qualitative study of the capabilities, opportunities and motivations to deliver oral health behaviour change messages to parents of young children in community settings

**DOI:** 10.1186/s12903-024-04344-0

**Published:** 2024-05-18

**Authors:** Joanna Goldthorpe, Lauren Kilbee, Iain Pretty, Sarah Cotterill, Jo Hart, Sarah Peters

**Affiliations:** 1 Manchester Centre for Health Psychology, Division of Health Research, University of Manchester, Manchester, UK; 2 Division of Medical Sciences, Colgate Palmolive University of Manchester Dental Health Unit, Manchester, UK; 3 Centre for Biostatistics, School of Health Sciences, University of Manchester, Manchester, UK; 4 Division of Medical Education, School of Medical Sciences, University of Manchester, Manchester, UK

**Keywords:** Primary care, Workforce, Paediatric, Behavioural science, Qualitative, Pandemic

## Abstract

**Background:**

The COVID-19 pandemic exacerbated vulnerabilities and inequalities in children’s oral health, and treatment activity virtually ceased during periods of lockdown. Primary care dentistry is still in the post-pandemic recovery phase, and it may be some years before normal service is resumed in NHS dentistry. However, opportunities to support the dental workforce through offering some preventative care in outreach settings may exist. This has the additional benefit of potentially reaching children who do not routinely see a dentist. The aim of this research was therefore to explore views around upskilling practitioners working in early years educational and care settings to support families of pre-school aged children to adopt and maintain preventative oral health behaviours.

**Methods:**

Using the Capability, Opportunity and Motivation model of behaviour (COM-B) to structure our data collection and analysis, we conducted semi-structured interviews with 16 practitioners (dental and non-dental) and analysed the data using deductive framework analysis.

**Results:**

The data were a good fit with the COM-B model, and further themes were developed within each construct, representing insights from the data.

**Conclusion:**

Early years practitioners can reach vulnerable children who are not usually brought to see a dentist, and have the capability, opportunity and motivation to support the oral health behaviours of families of children in their care. Further research is needed to identify training needs (oral health and behaviour change knowledge and skills), acceptability to parents, and supporting dental practice teams to work in partnership with early years settings.

**Supplementary Information:**

The online version contains supplementary material available at 10.1186/s12903-024-04344-0.

## Background

In the UK, the most common reason for children to go to hospital is to have decayed teeth removed under general anaesthetic [1]. This situation is costly, risky, painful, upsetting, and disrupts eating and sleep for children [2, 3]. In England, 1 in 4 children have tooth decay by the time they start school impairing their ability to engage successfully in the classroom right from the outset of their school career. In England, these rates are highest in the Northwest region, at 31.7% [4].

In England, the Chief Dental Officer advised on 25 March 2020 that all routine, non-urgent dental care should be stopped as part of COVID-19 infection control measures [5]. An estimated 365,000 infants (half of the birth cohort in the previous year), who would have been eligible for their first dental visit have been denied access to routine dental care. This is likely to have exacerbated an existing trend in low numbers of infants attending dental practices following onset of their first tooth; according to the Royal College of Surgeons, approximately 80% of one to two year olds did not visit a dentist for the year ending 31 March 2017 [6]. By early 2022, the capacity of dentists to see NHS patients remains reduced and many families are anxious about returning to their dentist for non-urgent assessments and treatment [7]. The impact on children who would normally have symptoms associated with tooth decay ameliorated by general anaesthetic could be devastating, and figures from the NHS Dental Referral Management System [8] show that this activity over the April-June 2020 lockdown was 75% less than that would normally be seen. This potential treatment backlog will produce a considerable challenge to secondary care when parents begin to bring their children to dental practices, especially considering these services were already experiencing a long wait time for some patients. Although some general dental practices provide early years preventative outreach services, these are dependent on local public health commissioning, interest, and capacity of local dentists. The dental profession has been affected by a persistent trend in the UK NHS of practitioner shortages and low morale [9], and this model is not consistently implemented and sustained.

Existing vulnerabilities and inequalities in the health of at-risk children and families [10] have been further amplified by the COVID-19 pandemic. Conditions that are exacerbated by lifestyle behaviours are likely to be triggered or made worse by the reduction in routine health promotion and preventative activities [11–13]. As dental practitioners focus on tackling a backlog of appointments for treatment, they may need to reduce time spent on preventative work and oral health promotion activity. Expanding and upskilling practitioners outside of the dental practice to support children and families to improve their oral health through encouraging behaviours such as brushing and reducing sugar intake may be beneficial. Furthermore, improving outreach in dental health promotion could be of continued and additional benefit beyond the pandemic recovery period for some of the most at-risk children and families who do not regularly engage with primary care dental services.

Involving parents and carers is crucial to the success of oral health interventions that target young children [14]. Child health behaviour is highly influenced by family context. For example, parent self-efficacy and action control are important determinants of toothbrushing supervision [15]. Hence, interventions directed at children should target parents and wider families. This has the added advantage of conferring benefits to siblings’ and parents/carers’ own oral health habits.

Our team has developed an online training programme to support dental practice teams’ conversations with parents about changing behaviours to improve the oral health of young children. Dental practitioners and students report that it improves their skills and knowledge, and is accessible and acceptable [16]. There is an opportunity to adapt this training so that it provides practitioners working in community settings with skills to have similar conversations with parent/carers of young children. However any adaptions require an understanding of the specific context within which the intervention would be implemented.

The COM-B model of behaviour is an established framework for exploring influences on health practitioners’ behaviour, such as incorporating behaviour change conversations into routine practice [17–19]. It encourages researchers to consider an individuals’ Capability (physical and psychological), Opportunity (physical and social) and Motivation (automatic and reflective) to perform a given behaviour (COM-B) [20]. It provides a useful lens through which to explore which community practitioners are best placed to deliver opportunistic oral health advice and behaviour change conversations with parents of young children.

The overarching aim of this study was to explore capabilities, opportunities and motivations of practitioners working in early years educational and care settings to support parent/carers of pre-school aged children (0–4 years) in the UK to have acceptable and useful behaviour change conversations about oral health.

## Methods

This study was approved by the University of Manchester Research Ethics Committee reference no. 2021–11084-17651.

The study design was qualitative and exploratory in nature.

### Recruitment

Participants were practitioners (dental and non-dental) working in community and outreach settings in a city in North West England with parents/carers of children aged 0–4 years. The local dental commissioner sent study information to all eligible practitioners using contacts from professional networks.

Participants were provided with written information about the study and had the opportunity to ask questions. Verbal informed consent was taken immediately before the interview took place. The researcher read the consent form aloud and participants provided a positive or negative response to each statement. Consent was audio recorded and stored as a record of informed consent.

### Participants

All participants worked in, or had good knowledge of, local dental outreach services:

Oral Health Improvement Officer^1^(*N* = 3); Dental nurse (*N* = 1); Dental Hygiene Therapist (*N* = 1); School Nurse (*N* = 1); Child minder (*N* = 2); Health Visitor (*N* = 1); Early Years Setting staff (Nursery Manager *N* = 1, Early Years Practitioner *N* = 2). As interviews commenced, participants suggested that we include some dental practitioners in our sample to explore their views on acceptability and safety of non-dental practitioners delivering preventative oral health messages. We therefore identified and recruited General Dental Practitioners (associate *N* = 2, principal *N* = 1) and a Community Dentist (*N* = 1). Of the total sample, 15 were female and one was male. Interviews lasted an average of 40.06 min (24–60 min).

### Procedure

Topic guides were used as prompts for semi-structured interviews and were informed by a review of relevant evidence and the COM-B model [21].

Interviews took place between April and October 2021 and were conducted by a researcher with over ten years of experience in community and public dental health research (JG). All interviews were audio-recorded and transcribed verbatim. Data were analysed using deductive framework analysis [22] based on the COM-B constructs [20]. This was followed by an inductive thematic analysis of the data that had been organised around individual COM-B constructs [23, 24] and was carried out to explore patterns in the data associated with each of the COM-B constructs. Each phase of the analysis was checked by another researcher (LK) for trustworthiness [21] and appropriateness of fit with the COM-B constructs (inter-rater reliability 75%).

## Results/ Findings

**Fig. 1:**
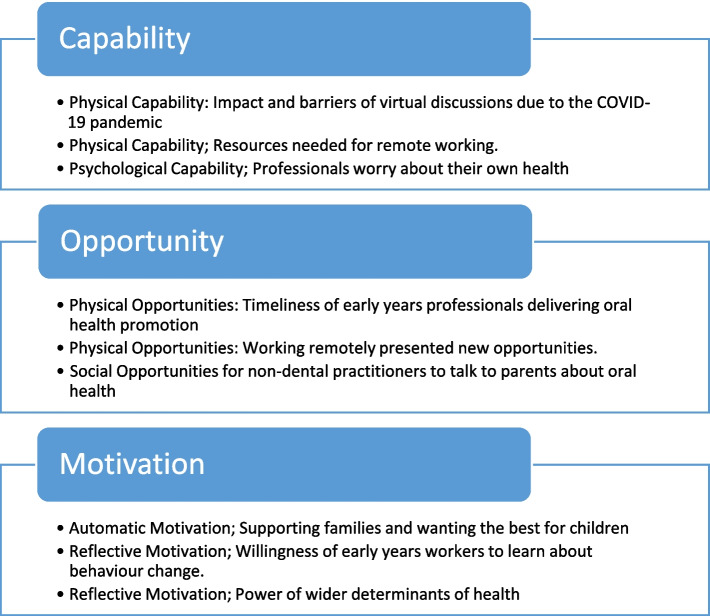
Findings from deductive and inductive analyses

Figure 1 shows the findings, presented under headings depicting the COM-B constructs, followed by sub-themes developed by organising data within each separate construct. Physical capability refers to an individual’s capacity to exercise the motor functions required to carry out a task in a specific environment. Psychological capability involves an individual’s ability to function well mentally in a specific environment. Physical opportunity refers to material and time resources required to carry out a specified activity and social opportunity refers to the necessary social and organisational support required to provide opportunity. Reflective motivation requires thought, planning and evaluation, and automatic motivation is driven by habits, instinct and inherent human drives.

### Physical capability

#### Impact and barriers of virtual discussions due to the COVID-19 pandemic

Although some participants continued to work with parents during periods of lockdown, capability to do this was impacted by the inability to see families in person and technical issues associated with virtual platforms:

*There are massive challenges with Zoom connections and things like that. So, you get parents that are embarrassed for you to look into the house, so they don’t like video calls. So, they prefer phone calls, a lot of them. And sometimes, they prefer you to phone them when the child isn’t about, so that they can really concentrate.* (P13, Dentist)

Despite the challenges, new but widely adopted ways of communicating remotely created some opportunities to reach families:

*It’s* [demonstrating toothbrushing] *not as effective over the phone. … But it works well for some patients like if, they’re not regular attenders, that might be your only chance at getting any advice across* (P04, Dental Hygienist)

#### Resources needed for remote working

Participants reported that additional resources were required to mitigate some of the barriers to delivering oral health promotion during the pandemic. This involved developing innovative visual tools and provision of resources usually accessed outside of the home to families who were isolating. Participants served diverse populations who spoke many languages, therefore, interpreters or bi and multi-lingual workers were vital to their ability to continue to work with non-English speaking families. Access to many of these resources were disrupted during COVID-19:

*I think it would be good for us to get some resources, like your toothpaste, toothbrushes, that sort of thing. When you used to go to the Sure Start Centres, they used to give them out so I think like we should be able to give them out as well. Because you can’t go to these centres anymore.* (P06, Child Minder)

### Psychological capability

#### Professionals worry about their own health

Participants were worried about infection and their own safety at work, particularly in the early stages of the pandemic where information about risks to and from children were unknown. Mitigating approaches included the provision of information and option to stay at home:

*…we have to think about our own health first. We have to think about what’s going on within that setting, and if we risk picking anything up, about taking that back to our families, and looking at what our families do, so that we’re not then passing anything onto them… So, we just kind of put a block on going in anywhere*. (P01, Oral Health Improvement Officer)

### Physical Opportunity

#### Timeliness of early years professionals delivering oral health promotion

During the pandemic, nurseries and childminders continued to care for the children of key workers, providing a consistent setting for the promotion of good oral health. Participants reported that the recent inclusion of supervised toothbrushing in the Early Years Foundation Stage curriculum presented a contemporaneous opportunity to engage early years professionals in delivering oral health promotion:

*With the new curriculum …oral health is part of it, where it never used to be. So, it's not a, maybe we'll do this, it's like this is part of the curriculum, whether you like it or not. We have to…we will provide the children with toothbrushes and toothpaste, and as long as COVID doesn't get worse, we will make sure that they do they their teeth at least once a day ... And we have to teach them how to do it.* (P09, Early Years Practitioner)

Participants felt that nurseries and childcare providers were advantageous settings for oral health promotion due to the relationships that staff build with families, the regular contact with young children and the potential this brings to encourage healthy oral health behaviours at an early age:

*A lot of other services, you are very limited aren’t you with the time slots, and the pressures of, you know you get 15 minutes to see a patient or whatever else, so… Some of these play groups, might be quite a good idea.* (P14, Health Visitor)

Working with families outside formal dental services, with professionals from different specialties provided opportunities to engage families that do not access typically dental services, and to initiate conversations about tooth-kind behaviours. Practitioners from different disciplines who already had relationships with families could support them to register with an NHS dentist, normalise attending the dentist, and potentially ameliorate dental anxiety:

*I think the way to overcome that [poor attendance at routine appointments] is by working closely with partners and I do think the dental practice maybe could work more closely with community partners whether it’s through us or through schools* (P03, Oral Health Improvement Officer)

#### Working remotely presented new opportunities

Despite the pandemic-related disruption to delivering in-person oral health care, remote working offered opportunities to deliver preventative work, including behaviour change conversations outside of the dental practice using video technology. Participants noted how well school-aged children engaged with practitioners online:

*I was a bit of a sceptic at first… I think it’s been really interesting how children engage via video. I thought they’d just, kind of, be ignoring me, but they’re not. They’re, like, getting up and they’re trying to show me their teeth, and I’ve been doing a little bit of tooth-brushing on that sometimes, and they’ve been getting right up close to the camera.* (P10, Dentist)

Although treatment activity in dental practices continues to be highly restricted due to infection control practices, remote working provided scope for delivering preventative messages in a more informal setting and making clinic visits more efficient:

*I’ve done more telephone and video consultations about prevention. So that when they actually come into the clinic, they can just have the treatment done, rather than the long preventive conversations that we have with the parents. And actually, that’s worked a lot better because it gives you a bit more time when the parents aren’t stressed because the kids aren’t kicking off and throwing things around the surgery…they’re able to ask questions and I’ve had their full attention*. (P13, Dentist)

### Social opportunity

#### Opportunities for non-dental practitioners to talk to parents about oral health

Many social opportunities existed for non-dental practitioners to have oral health conversations with parents. Early years practitioners working in nurseries and children’s centres and childminders working from home were cited as having particularly good relationships with parents. Conversations that might be deemed difficult or delicate could take place in private and there were multiple opportunities for practitioners to pick up on cues, leading to opportunistic behaviour change conversations:

*…some children will struggle, like trying different tastes, or eating certain foods, so maybe that’s a way in. Say, … “well, do you talk to your child about how it is good to have a healthy mouth?” “And then if we’ve got a clean mouth, we can try these new tastes and things that have different textures.”* (P11, Early Years Practitioner)

### Automatic motivation

#### Supporting families and wanting the best for children

Participants who worked in childcare settings felt that engagement with training around oral health prevention and having behaviour change conversations would be something that was part of their role in supporting children. Therefore this was an extension of their *in loco parentis* position. They saw their role as involving caring tasks that support children’s routine at home (such as toothbrushing), which parents may have not been able to support themselves. Participants wanted to help children and improve outcomes for families generally:

*We, in the past, have had families where one sibling after another has had to go for a general anaesthetic. So, you would hope that with those behaviour changes, and if they’re actually supported and reviewed, that one of the impacts would be that it didn’t pass down to each sibling, and another one would hopefully be that, you know, that behaviour change is permanent*. (P03, Oral Health Improvement Officer)

Outcomes were perceived broadly, and included improvements in general health, reduction in the social and financial opportunity costs of poor childhood oral health that can burden families, and cost savings and service improvements for the NHS:

*We are all interlinked...I do think long-term we would see the impact of this, not just from an NHS financial perspective, but things like waiting lists. But most importantly the benefits that that individual’s going to have. So, that child’s not going to suffer from dental anxiety because they’ve had teeth extracted as young as five or ten. That they take on responsibility for their own health as well and parents take on that responsibility for younger children who are dependent.* (P12, Dentist)

### Reflective motivation

#### Willingness of early years workers to learn about behaviour change

All participants felt that supporting oral health was part of a wider set of responsibilities and obligations required by their professional role. Furthermore, they reported being accustomed to accessing continuing professional development, and most felt motivated to upskill to improve their conversations around oral health behaviour change. Some participants with responsibility for target setting and evaluation recognised that undertaking training to support specific skills such as behaviour change could support wider professional aims and identification of key goals for their team.

A smaller number of participants were concerned that parents would not engage in behaviour change conversations or might be reluctant to implement advice or techniques offered. The perceived lack of motivation in their patients was cited as a de-motivating factor in the professionals who worked with them, and participants felt unskilled to help motivate these families:

*I find it hard, because a lot of the groups that I work with, they’ve no interest in the baby teeth at all. …you’ve got one child after another in a family, that are having a GA, and they think it’s normal. And it’s really shocking. And then you’re explaining things, you know, and they’re like, well, as long as the adult teeth look nice, we’re not bothered.* (P08, Dentist)

Nevertheless, participants reflected that, regardless of their doubts that oral health promotion and support with behaviour change would always have a positive effect, it was important to continue to engage patients in the hope that some families would benefit:

*…you always have those parents or carers who will, like, be very resistant and any suggestion that they’re doing anything that isn’t quite up to standard, they take total offence, you always get that,...But, you know, for every one, you might get ten people or 20 people or 30 people who might be very responsive to advice given the right way*. (P13, Dentist)

#### Power of wider determinants of health

Although many participants reported feeling motivated to have preventative oral health conversations with families, they were cynical about the degree to which they could yield positive outcomes in the face of wider contextual and environmental barriers. These included poverty and related family stress, targeted advertising to children and families, ubiquitous sugary foods and ambiguous labelling:

*…still looking at what’s marketed as healthy things like a cereal bar… I think it’s very confusing generally. I mean, even look at the, like, No Added Sugar Vimto, and everybody says they drink that, to me. And I have to say to them, “well, it’s still got the sugar in it”.* (P10, Dental Associate)

Similarly, some participants reported that the ability of many families to implement change was impacted by their socio-economic conditions. Oral health issues often overlapped with other issues that needed to be addressed and this was particularly evident in families referred to Early Help (a formal social support intervention). Participants felt that some families needed holistic support with wider issues before oral health could be addressed:

*Children have told us they do not have a toothbrush at home, and they have never brushed their teeth.* (P09, Early Years Practitioner)

## Discussion

We found the COM-B framework useful for exploring participants’ views on the potential for successful oral health behaviour change conversations with parents of young children to take place in outreach settings.

It is perhaps unsurprising that participants focused on physical and psychological capability for them and their colleagues to have behaviour change conversations in the context of the COVID-19 pandemic. Established routines had changed both for families and practitioners and normal ways of working were particularly affected for dental practice teams in ways that were out of their control. Participants from all professional groups reported worries and uncertainties about infection risk as affecting their psychological capability to deliver services.

Nurseries and childminders continued to provide childcare during the pandemic for key workers’ children. This provided physical and social opportunities to have continued, albeit reduced, contact with parents, for example, online and at socially distanced pickups and drop offs. Participants from a variety of backgrounds reported specific physical opportunities for early years practitioners to have oral health behaviour change conversations with parents due to recent changes to the early years foundation stage curriculum [25], which included supervised toothbrushing from September 2021. This offered opportunities for practitioners to introduce conversations about oral health behaviour change and provided potential teachable moments for both staff and parents. In addition, practitioners could capitalise on social opportunities at pickup and drop off and regular reporting on children’s progress to talk to parents about oral health.

Participants working in early years settings reported having automatic motivation to care for children in many ways, including supporting their health; this was cited as an intrinsic part of their roles. Although many participants reflected that supporting families to mitigate the wider determinants of health could be a motivating feature, some felt overwhelmed and unable to make a difference. This resulted in them becoming de-motivated and cynical about their ability to support oral health behaviour change.

Provision of information alone is not sufficient to bring about meaningful behaviour change in children and families, and successful interventions for oral health should draw upon on behavioural and psychological science. [20, 26]^.^ Psychological theories are important in understanding health behaviours and in developing effective interventions to support oral and dental health-related behaviour change [27, 28]. Training healthcare professionals in theory-informed behaviour change interventions is acceptable in a range of settings such as midwifery [29], general practice [18] and mental health care [30], in addition to dentistry [31]. The COVID-19 pandemic-related issues outlined in the introduction to this paper have contributed to a situation where a 2022 British Dental Association survey estimates that 9 out of 10 dentists offering NHS provision were not able to offer appointments to new adult patients, and over half of children in England do not have access to an NHS dentist [32]. Identifying a setting where oral health behaviour change conversations with families can be acceptably delivered could form an important part of a preventative strategy for childhood dental disease.

In summary, our findings indicate that practitioners working in early years settings have the capability, opportunity and motivation to have oral health behaviour change conversations with parents. This view is supported by other professionals including, crucially, the dental professionals who participated. This is an important finding as dental professionals need to trust and support non-dental professionals to provide adequate, non-harmful prevention advice for enhanced outreach services to be successful. As many early years settings begin to introduce supervised toothbrushing, there are opportunities to relay advice and provide support to parents, however the workforce will need training in the best way to do this. Our findings suggest that provision of this training is acceptable.

Training a non-dental workforce, particularly those operating in more deprived areas who have opportunities to access and influence families who do not regularly take their children to the dentist, has potential to address oral health inequalities in the post-pandemic period and beyond. Oral health practitioners could provide some preventative care to those children most in need but least likely to receive it, and encourage parents to bring their children to appointments at primary care dentists. Dentists with NHS contracts could additionally facilitate this through advertising availability and offering places to these families in partnership with early years care providers. A joined-up approach to getting families most in need into dental practices is needed.

A strength of this research is the inclusion of practitioners from a variety of community and outreach settings, allowing them to express their insights and views in depth using interviews. Although qualitative research does not seek to make population-level generalisations, our sample was drawn from a specific cohort located in one geographical setting (North-West England) and their views may not be representative of the wider healthcare practitioner population. The participants we recruited worked in, or had good knowledge of, local dental outreach services, therefore our sample represents the views of professionals who are already engaged in and knowledgeable about the topic. We were only able to recruit one male to this study and therefore our data represents mostly female professional views.

## Conclusion

In conclusion, early years childcare providers are well-placed to deliver preventative oral health care to children most in need and could potentially support families to develop and maintain healthy behaviours to support good oral health. Further research is however needed into initial training requirements including content, preferred mode of delivery and continuing professional development. In addition, supportive relationships with NHS dental teams are needed to ensure families receive the right information and can access treatment and specialist care from dental professionals in a timely and accessible manner.

### Supplementary Information

Supplementary Material 1.

## Data Availability

The datasets used and/or analysed during the current study are available from the corresponding author on reasonable request.

## Footnotes

1 The role of an oral health improvement officer is to promote oral health in children in outreach settings (Children’s Centres, nurseries, schools) from 0–16 years. In this context, oral health improvement officers are part of a public health team and not employed by general dental practices.
