# Supplementary material for: Supporting post-pandemic recovery: a qualitative study of the capabilities, opportunities and motivations to deliver oral health behaviour change messages to parents of young children in community settings
Source: BMC Oral Health. 2024 May 18;24:580. doi: 10.1186/s12903-024-04344-0 (PMC11102627; doi:10.1186/s12903-024-04344-0)
Supplement: Supplementary file 1 — Supplementary Material 1. [file 12903_2024_4344_MOESM1_ESM.pdf]

Version 1 12/10/2020

The POLAR BEAR Study (Covid-19 pandemic): Topic Guide for dental care practitioner interviews

As this is a semi-structured interview, the questions listed below will serve as prompts for the interviewer. The exact wording and order of the questions may vary as the interview progresses, and some further questions may be asked in order to pursue emerging ideas relevant to the research question. The following interview schedule will act as a guide for the researcher whilst conducting the interview. Bullet points below questions show suggested prompts.

Training

Brief the participant on the POLAR training: content, aims, evidence.

1. ***How useful would you find the POLAR training?*** *(Capability)*

- How confident are you in having behaviour change conversations with parents?
- Would learning new techniques help you to support parents?
- How confident might you feel after training?

2. What would you like to learn more about? Why? (Capability)

- How to pick up on cues/ introducing "delicate" topics
- BCTs
- What techniques do you use (if any)? What, if any, do you think are useful for working with parents? Other patients?

3. ***What contact do/will you have with parents (Opportunity)***

- In their homes
- In other settings e.g. schools, dental practices
- Online

4. What are the challenges and opportunities for having behaviour change conversations during lockdown and Covid-19 related restrictions? (Opportunity)

- What work can you/ have you done?
- Issues around delivery
- Issues around safety
- Families spending more time at home
- Parent/ child acceptability

5. Have you managed to deliver oral health promotion activities during lockdown and Covid-19 related restrictions? If so, please tell me about a time when you have done this. (Capability/opportunity)

- What did you do
- How did you deliver it

- Parent/ child reaction
- Probe for occasions when it went well/not so well

6. What role or services are best placed to support parents with establishing or maintaining health related habits in general during lockdown or Covid-19 related restrictions and why? (Capability/opportunity) probe for health visitors, oral health promotion team, dental practice teams, school nurses

- Trustworthy/ credible/ knowledgeable
- Convenient
- Resources to deliver support online/ face to face with safety restrictions
- Low or no cost

7. *What might the effects on children's oral health be due to dental practices closing under lockdown and what might the impact be? (Motivation)*

- Pain – sleeplessness, difficulties concentrating, low mood
- Other symptoms e.g. bleeding gums, difficulties eating
- Waiting for treatment
- Not changed

8. *What do you think could be the impact of having behaviour change conversations with parents of at risk children during lockdown/ restrictions*? *(Motivation)*

- Improved oral health of the child
- Improved oral health of the household
- Supporting stressed parents
- Transferable skill to other behaviours

9. *What do you think could be the impact of Polar training training on you/ the service you work for ?* (Motivation)

- Need?
- Transferable skills
- More confidence

***10. What might the public health benefits of you/ other practitioners undertaking this kind of training be both during and after the Covid-19 pandemic?* (motivation)**

- Reduction in child extractions under GA
- Reduced costs to the NHS
- Contact with families who don't attend regular dental check ups
- None
- Healthier households

11. What other resources and support might you need? (Opportunity)

- Time
- Equipment (Computer, phone)
- More training
- PPI

12. Is there anything else you would like to add regarding this topic that we may have missed?
